# Health care experiences of individuals accessing or undergoing *in vitro* fertilization (IVF) in the U.S.: a narrative review of qualitative studies

**DOI:** 10.3389/frph.2025.1490917

**Published:** 2025-02-13

**Authors:** Summer K. Peterson, Larissa Jennings Mayo-Wilson, Lauren Spigel, Isabel Morgan, Adriana Parker

**Affiliations:** ^1^ Department of Health Behavior, University of North Carolina, Gillings School of Global Public Health, Chapel Hill, NC, United States; ^2^ Department of Maternal and Child Health, University of North Carolina, Gillings School of Global Public Health, Chapel Hill, NC, United States

**Keywords:** *in vitro* fertilization, IVF, qualitative, narrative review, United States, health care

## Abstract

**Background:**

*In vitro* fertilization (IVF) is an increasingly common method of assisted reproduction given the high rates of infertility in the United States (U.S.). However, despite growing utilization of IVF technologies, there is little known about the experiences of those accessing or undergoing IVF, particularly among adults in the U.S. The aims of this review are to (1) explore how economic, emotional and physical health, and interpersonal relationships impact and are impacted by accessing or undergoing IVF, and (2) understand the role of healthcare providers and the healthcare system.

**Methods:**

A narrative review was conducted to summarize the current literature and provide insight into potential channels for care improvement. Eligible studies were published in English from 2013 to 2024 which qualitatively evaluated experiences of individuals and couples accessing or undergoing IVF. Peer-reviewed publications were identified from three electronic bibliographic databases. Methodologic rigor was assessed by two reviewers who also abstracted data on the study's characteristics as they pertained to four domains: health systems, economic, interpersonal, and physical and emotional health. Among the 32 papers retrieved for review, 22 met inclusion criteria and were retained for analysis.

**Results:**

The available literature suggests accessing and undergoing IVF can be positively and negatively influenced by health systems, economic, interpersonal, and physical and emotional health factors. Often an individual or couple experiences multiple factors that compound to create a complex situation. Health systems-related factors included physician interaction and challenges with information volume and processing. Economic challenges primarily pertained to financing IVF and navigating insurance coverage. Interpersonal factors related to changes in relationships with partners, family members, and friends due to IVF. Physical health concerns (e.g., pain) and emotional health concerns (e.g., sadness, stress) were also noted by all included papers.

**Conclusions:**

Efforts to improve care experiences of adults accessing or undergoing IVF are urgently needed. The evidence base points to a need for provider sensitivity trainings, clinic-based intervention, and community education in both physical and virtual spaces.

## Introduction

Over the past decades, *in vitro* fertilization (IVF) has become an established and effective medical treatment for various forms of infertility (1)—in partial response to the demographic trend of delayed parenting in the United States (U.S) (2). Infertility is defined as an inability to attain a successful pregnancy after timed unprotected intercourse or therapeutic donor insemination for 12 or more months (3, 4). Approximately 19% of married women aged 15–49 years in the U.S. experience infertility (5), resulting in roughly 7 million U.S. couples seeking care for infertility each year (6). The number of pregnancies that are conceived via IVF in the U.S. increases annually, representing 79,942 live births stemming from IVF in 2020 or 2.2% of all births in the U.S (7, 8). An IVF treatment cycle commonly includes ovarian stimulation, the retrieval of oocytes, and fertilization of collected oocytes and embryo culture, followed by the resultant embryos being transferred to the patient's uterus for immediate conception and/or being transferred to cryostorage for later use, and progesterone supplementation (1, 3, 4, 9). IVF patients and their partners undergo these medical treatments and required monitoring at varying pace through the health care system (10). An IVF cycle is complete once all frozen and/or fresh embryos have been transferred (3, 4, 9).

Qualitative research elicits beliefs and opinions about people's lived experiences using their own words and can provide rich contextual insight to inform clinical practice (11). Yet, despite increasing utilization of IVF in the U.S. and a growing recognition by the assisted reproduction community to understand IVF care practices, there is limited qualitative evidence regarding U.S. patients’ IVF care experiences. Most U.S.-based studies of IVF-treated patients have centered on quantitative clinical outcomes relating to pregnancy and childbirth (12–15). On the other hand, most qualitative studies with patients and providers at fertility clinics have been conducted outside of the U.S., predominately in in Europe (10, 16–22), Asia (23–27), Australia (28, 29), and some regions of Africa (30).

Qualitative research outside of the U.S. has shown that IVF patients and their partners face a number of challenges. Undergoing IVF treatment can involve pain from the hormonal injections and emotional distress, such as anxiety, sadness, or depression, in anticipation of or after a cycle fails (16, 22, 25, 28, 30, 31). Patients must also manage disruptions to their daily lives from treatment appointments and cope with the financial stress of affording the resources they need for treatment (16, 17, 25). IVF couples undergo an average of 2.7 cycles, spending $61,377 USD out-of-pocket, to achieve a live birth (32, 33). In addition, despite technological advances, success rates of IVF are moderate. Although a total of 326,471 IVF cycles were carried out in the U.S. in 2020 (7, 34), only 23% resulted in a live birth (7). Thus, the majority of IVF patients must consider other pathways to parenthood, such as foregoing their desires for parenthood or adoption. At increasing costs, patients may repeat IVF treatments for months or years prior to pregnancy or prior to discontinuing attempts of biological parenthood. This has resulted in some patients voicing frustration about fertility clinics providing overly positive false hopes regarding their chances of success (18, 28, 35).

Despite the abundance of qualitative research in other countries, little is known about U.S. women's IVF care experiences in their own words. Questions remain about qualitative themes relating to *health systems experiences*, such as how patients select fertility clinics, their perceptions of how providers present IVF information and subsequently discuss procedures and chances, their views on how clinics support them through treatment challenges, or quality and continuity of care (16–18). Questions also remain about themes relating to *economic experiences*, such as how patients determine expected IVF care costs and the perceived impact of IVF expenses on households (10, 36). Only 15 out of 50 states (∼30%) mandate private insurance coverage for some infertility treatments and only four states (e.g., IL, MA, NJ, RI) offer comprehensive mandated health insurance for IVF (up to 4 IVF cycles) (13, 14). Further, only one state (e.g., NY) mandates Medicaid coverage for fertility services, specifically fertility testing and fertility medications, while no state Medicaid program provides coverage for intra-uterine insemination (IUI) or IVF (14, 37). Information is also lacking on the *physical and emotional experiences* of IVF patients, such as coping with time requirements, pain, or anxiety, or *interpersonal experiences*, such as how patients decide to continue or end treatment, how they discuss IVF with others, or how they adjust to parenting or non-parenting outcomes (38). A deeper analysis of existing qualitative IVF studies in the U.S. could help to develop appropriate interventions and policies to improve patients’ experiences during assisted reproduction. Ultimately, reducing infertility-related stressors could improve maternal and child health outcomes of biological and adopted children (22, 39–41).

This study aimed to explore the following research questions: (1) How does IVF impact the financial, emotional, and physical wellbeing of women who access or undergo IVF treatment or consultation? (2) What is the role of the healthcare system, including fertility clinics and providers, in the overall experience of accessing or undergoing IVF treatment or consultation? (3) How does accessing or undergoing IVF impact interpersonal relationships? This study reviews what is known about IVF care experiences in the U.S. among studies using qualitative data generated from women undergoing, planning to undergo, or having undergone IVF. To our knowledge, this study is one of the first narrative reviews of qualitative studies examining IVF care experiences in the United States. Findings are intended to summarize the state of the qualitative evidence and to provide insight on potential ways to improve clinical and community-based IVF practices.

## Methods

### Search strategy

Table 1 lists the search topics, terms, and parameters used in the narrative review. We aimed to identify *in vitro* fertilization studies that involved qualitative research methods. Three electronic bibliographic databases were used. These were: MEDLINE/PubMed, PsycINFO, and Excerpta Medica Database (EMBASE). These databases were selected to cover a broad range of disciplines, including medical, public health, psychological, and social science research. The search terms included words related to the review's topic (e.g., IVF) and study design (e.g., qualitative) and included synonyms and spelling variations, where applicable (Table 1).

**Table 1:** Search strategy for electronic databases.

MEDLINE/PubMed, EMBASE, PsycINFO			
Search topic	Reproductive technology		Study design
Selected search terms	*in vitro* fertilization; IVF; reproductive technology; infertility	and	Qualitative; exploratory; interview(s); focus group(s); formative
Other search parameters	Publication date of 2013–2024 Publication language of English		

### Inclusion criteria

Studies that met the following criteria were included: (i) research on women who had experience with IVF consultation or treatment; (ii) research that reported qualitative findings of IVF experiences; (iii) research that was performed in the United States (U.S.); and (iv) research that was published in English over the last decade, between 2013 and 2024, up to the time of publication of this review. Exclusion criteria included non-research publications (e.g., chapters, commentaries, protocols, editorials, conference abstracts), studies published in languages other than English, studies conducted with populations outside of the U.S., and studies that lacked qualitative findings relating to IVF.

### Title, abstract, and article screening

**Figure 1:**
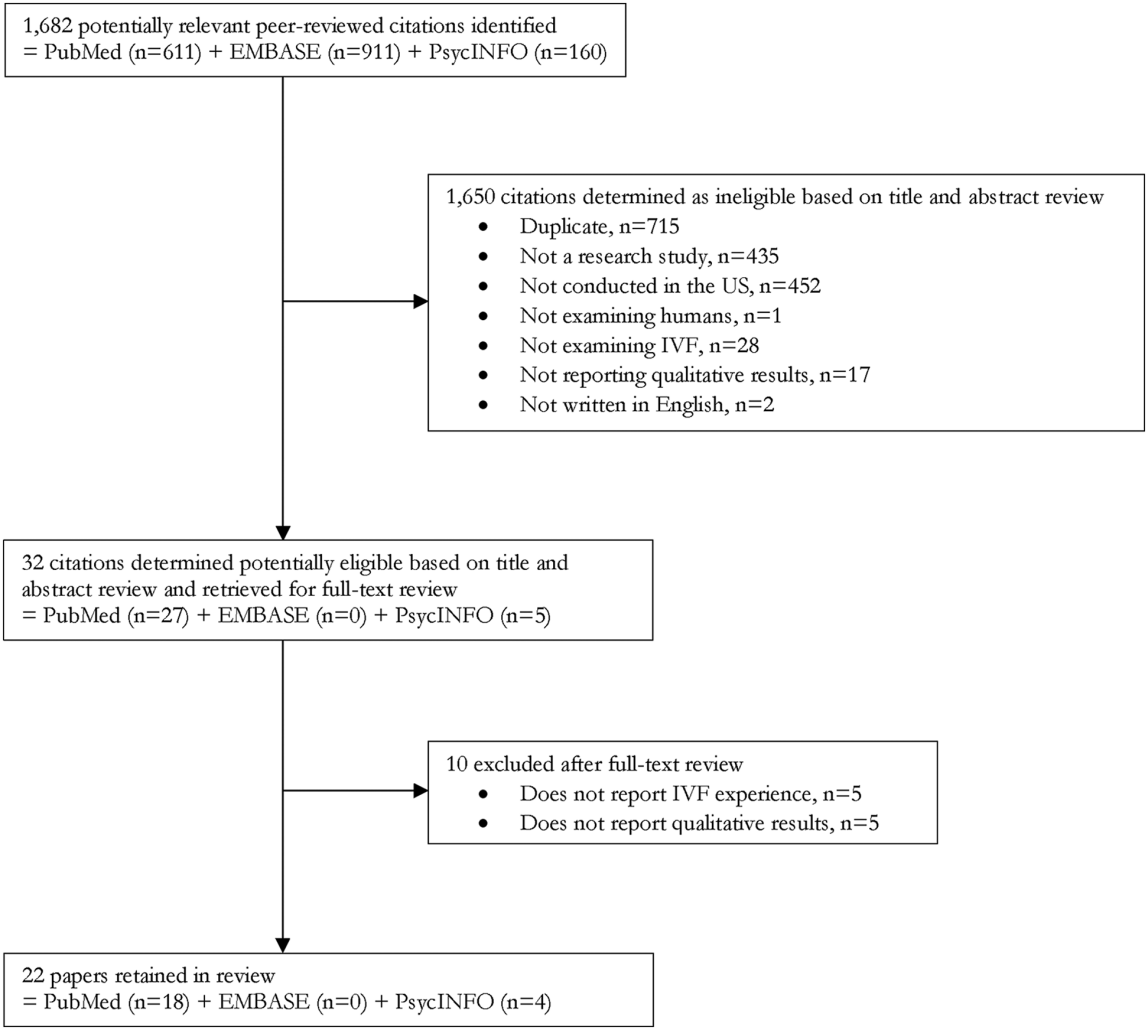
Flow diagram of search and screening results.

Figure 1 illustrates the search and screening process. Once the search terms were applied to the electronic databases, potentially relevant citations were selected based on the screening of the title and abstract by the primary reviewer (e.g., the first author), who then downloaded the full-text publication. All full-text publications were then reviewed by two reviewers (e.g., the first and second or third authors) to further assess eligibility. Each reviewer independently excluded publications not meeting the inclusion criteria. Discrepancies in study eligibility were discussed and corrected based on consensus. Once this process was completed with MEDLINE/PubMed, the reviewers began the process again with PsycINFO and EMBASE databases and extracted and reviewed potentially relevant citations that were not duplicates. All citations were entered into an Excel database to track the retrieval process and document reasons for inclusion or exclusion.

### Study appraisal

A final set of selected articles underwent a systematic appraisal by two appraisers (e.g., the first and second or third authors) in addition to a rapid review by an *ad hoc* reviewer (e.g., the second author) (Table 2). Data were extracted and documented in a study appraisal form for the following items: article identification number, author, year of publication, title, search strategy source, publication journal, study design, study objective, sample/participants, and key findings across four assessment domains: (i) health systems findings, (ii) economic findings, (iii) interpersonal/social findings, and (iv) physical and emotional findings. Data were also extracted on suggestions made by the publications’ authors relating to strategies to improve IVF care. Each publication's two appraisal forms were compared and used to synthesize and write the review's results.

**Table 2:** Summary of publication characteristics and research findings by domain for selected studies (*N* = 22).

First Author (Year)	Qualitative study aim	Qualitative study design	Qualitative sample	Key findings *(Assessment Areas)*				Author suggestions to improve IVF support
				Health systems	Economic	Interpersonal and social	Physical and emotional	
Anguzu et al. (2020) (42)	To examine couples’ decision-making for infertility treatment	Longitudinal in-depth interviews	=34 opposite sex couples (68 total people) from the Midwest with a reproductive specialist consultation	Patients used IVF literature from clinic to understand treatment process.	Finances and lack of insurance were a constraint with ability to secure IVF quickly if had high income.	Careful to discuss IVF plan, changes, or risks with absent partner or others to avoid added stress.	Hesitancy to share emotional distress regarding failed cycle or failed pregnancy with partner.	Recommended both partners be present at clinic visits and clinics to provide more information on IVF options and costs.
Borowczak and Rotoli (2022) (43)	To examine social support experiences among adults reporting infertility	One open-text question added to a quantitative survey	=110 adults across the US experiencing infertility who visited a fertility support group webpage	Clinicians were helpful, but little known of personal chance of success. Amount of clinical info and inquiry was overwhelming.	ND	Friends and family have limited guidance or ability on how to help if haven't had IVF.	Contributes to lower self-confidence and spousal or partner relationship strain. Reluctance to tell friends.	Noted clinicians avoiding infertility assignment to either partner to reduce blame. Recommended sharing IVF success stories and fears.
Buechel et al. (2022) (44)	To understand how military personnel navigate fertility services in military health system	Cross-sectional in-depth interviews	=32 fertility patients, partners, and providers from six military treatment facilities	Time constraints in navigating IVF within of military schedule. Welcomed locations with clinics given challenge of transferring embryos.	Patients saved and obtained interest-bearing loans to pay for IVF. Costs varied substantially by state or location.	Received more support from women or from supervisors and peers with IVF experiences.	Emotional distress re: impact of fertility treatment on career. Unable to rest at home during discomfort or fatigue.	Advocated for IVF policy information and terminology given to employers and to remove infertility diagnosis and partner requirement.
Ceballo et al. (2015) (45)	To assess experiences of getting pregnant, including infertility treatment-seeking	Cross-sectional in-depth interviews	=50 African-American women from the Midwest experiencing infertility	Clinics suited for those with schedule flexibility. Welcomed doctor referrals to counseling. Often felt loss of control in clinical decisions.	Clinicians assume inability to pay due to race/class. Challenging to absorb costs or use most of savings on IVF.	Described concerns of whether race, class, and/or gender influenced their interactions with others.	Affects self-esteem and femininity. Prefer not to tell partner or peers due to privacy and distress.	Recommended sharing information to reflect diverse racial, socio-economic IVF patients to increase support and to minimize stereotypes.
Cebert-Gaitors et al. (2022) (46)	To characterize facilitators and barriers to infertility treatment	Cross-sectional in-depth interviews	=13 African-American women from the DC metropolitan area who had infertility treatment	Patients relied on online reviews and demographic data to select a fertility clinic.	Reported limited knowledge of financial options.	Discussions with others assumed no treatment need. Avoided receiving unsolicited advice.	Limited assessment of patients’ anxiety or depression during IVF process.	Called for more support of infertility-related anxiety and earlier referral infertility concerns.
Cusatis et al. (2023) (47)	To examine decisional regret years after a fertility consultation	Open-text questions added to a quantitative survey	=120 adults from the Midwest who had one or more infertility consultations	Patients expressed feeling misled by the provider re: viability of treatment options.	Disappointment and unhappiness was expressed regarding cost of adding a child to the family.	Described the added stress from infertility treatment on romantic relationships.	Concerns around IVF and long-term health expressed by partners of the carrying person.	Highlighted importance of offering patients and partners psychosocial support through decision making processes.
Ferland and Caron (2013) (48)	To examine experiences managing infertility	Cross-sectional in-depth interviews	=12 post-menopausal women from New England who experienced infertility	Difficulty managing clinician's insensitive language (“kill the stork”) and decisions to end IVF due to impact on clinic's failure rates.	Patients discontinued IVF due to high credit card debt from unsuccessful cycle. Bank loans are often denied.	Noted couples’ discussions to prioritize quality of life over on-going IVF, despite negative feedback from relatives.	Reported feeling guilty and not enjoying sexual intercourse due to infertility concern. Painful procedures felt experimental.	Suggested clinical referral to counseling for patients to cope with treatment and training to IVF peers about what (not) to say or do.
Gentile (2013) (49)	To evaluate infertility treatment experiences and decisions to stop	Cross-sectional in-depth interviews	=9 adults who sought ART	Discontinuity in providers, rushed decisions, and repeated attempts to receive care for pain.	Difficulty rationalizing prior IVF spending relating to gains (or losses).	Discomfort in discussing with others. Trauma from others who achieve pregnancy.	Additional distress when pain and discomfort were minimized by others.	Highlighted importance of training in cultural competence and care quality among providers.
Lee (2019) (50)	To examine infertility awareness, diagnosis, and management	Cross-sectional in-depth interviews	=54 women across the US experiencing infertility	Clinics were source of IVF info with unexpected and late diagnoses that limited care options.	Regret in omitting financial/monetary investment or past spending to preserve eggs.	Little prior discussion on possible infertility treatment. Burden to educate others.	Reported experiences of frustration and anger.	Recommended having infertility talks earlier to better plan childbearing with career goals.
Leyser-Whalen et al. (2018) (51)	To assess process of self-identifying as infertile	Cross-sectional in-depth interviews	=28 adults from the Midwest who had used any fertility treatment in past five years	Individuals amass many clinic visits and diagnoses not always aligned with self views of (in)fertility.	ND	Reported desire to relieve partner of treatment stress or depression given coping ability.	Described worries regarding whether infertility was due to or causing unhealthiness.	Suggested increased dialogue regarding infertility criteria, diagnosis meaning, and treatment options.
Leyser-Whalen et al. (2021) (52)	To evaluate care perceptions of generalist and specialist fertility providers	Cross-sectional in-depth interviews	=28 adults from the Midwest who had used any fertility treatment in past five years	Initial contact is by woman with PCP or OB/GYN rather than reproductive endocrinologist to have less costly care.	Concern re: clinics being motivated by profit to obtain paying patients, even if cycle success is unlikely.	Patients frustrated by little listening and dismissed urgency in communication regarding fertility.	Reported experience of receiving “rough” care which caused increased pain and distress.	Highlighted need for staff sensitivity training for all genders and offer of counseling.
LoGuidice (2022) (53)	To assess IVF experiences of survivors of sexual abuse	Cross-sectional in-depth interviews	=3 women from New England who had undergone one IVF cycle	Called for trauma-informed care among fertility providers, including use of sensitive language.	ND	ND	Expressed being unprepared for IVF pain, grief, discomfort, and side effects.	Recommended more trauma-informed training of providers and inclusion of patients in decisions.
Mac Dougall et al. (2013) (54)	To examine later-life parenting after assisted reproduction	Cross-sectional in-depth interviews	=61 women from CA who had undergone IVF and given birth after age 40	Skepticism re: clinical IVF statistics’ applicability to own fertility. Included self-referral and OB/GYN referrals.	ND	Women recalled social networks noting how easy it is to get pregnant with little note of infertility issues.	Expressed surprise, alarm, and dismay for treatment need given assumption of fertility.	Advocated for earlier and more comprehensive fertility education to all genders to inform childbearing decisions.
Mayette et al. (2024) (55)	To understand how patients seek information during ART care	Cross-sectional in-depth interviews	=15 female ART patients	Expressed difficulty understanding materials but preferred those from providers over other academic resources.	Expressed difficulty understanding the cost and worry about quality insurance coverage.	Social media was a helpful tool for connection and information sharing, though misinformation was a concern.	Described wishing they pursued mental health support, but not knowing about support options.	Suggested provision of extra resources from providers to dispel misinformation and connect patients to mental health tools.
Osadchiy et al. (2020) (56)	To examine experiences of male infertility care-seeking	Open-text posts extracted from online discussion board	=97 online posts from 73 male infertility online discussion board users	Relied on social media network to interpret medical/lab test results while waiting for clinic visit.	ND	Appreciated shared IVF info from other users, although difficult to vocalize sad feelings to partner.	Described feeling stressed, emasculated, and depressed given diagnosis and uncertainty.	Suggested enhancing provider communication for male infertility with complementary online support groups.
Öztürk et al. (2021) (57)	To understand effects of infertility treatment	Cross-sectional in-depth interviews	=12 women across the US experiencing infertility	Nurses invalidated infertility concerns, withheld diagnostic tests, or ignored pain. Provided discordant information. Stress at specific venues.	Financial costs were a treatment obstacle with stress from insurance policies. Women sold belongings to cover IVF costs.	Beneficial to have therapy, counseling, and online support groups for social support.	Challenges in managing loss of sleep, nausea, stress, anxiety, hair loss, mood swings, and pain caused by IVF protocols.	Recommended more informational and emotional support (e.g., empathy and compassion) be given to patients for social circles and providers.
Palmer-Wackerly et al. (2019) (58)	To examine provider communication and continuity of fertility care	Cross-sectional in-depth interviews	=25 adults experiencing infertility	Patients more likely to continue IVF care if clinicians offered individual care plans and validated grief, treatment goals, and physical limits.	Families used loans and savings to pay costs. Skeptical of clinic's profit motives and if IVF spending was wasteful.	ND	Described emotional (anger, fear, sadness) and physical toll (pain) from treatment rounds.	Advocated for better communication of all treatment options, including risks and benefits. Suggested emotional support, avoiding false hopes.
Perone et al. (2020) (59)	To assess trends in social media topics on ART among women undergoing treatment	Open-text posts extracted from online social media	=209 online posts from women at varying stages of IVF	Covid protocols limited partner accompaniment to ART clinics. Some women obtained advice online to select an ART clinic.	ND	Detailed social support from family, religious, and community members, many of whom had prior IVF experiences.	Positive, negative, and conflicting emotions. Included changes to body from IVF and setbacks from cycle cancellations.	Suggested formalizing online social support groups to improve community-based IVF care.
Perone et al. (2021) (60)	To assess IVF patients’ experiences	Open-text posts extracted from online social media	=452 online posts from women at varying stages of IVF	IVF clinical process often interrupted to address other reproductive health issues (cysts, fibroids, polyps, miscarriage).	Financial strain not only for IVF but also other methods to enhance fertility (supplements and acupuncture).	Posts consisted of women educating others on specific IVF protocols and tracking IVF milestones.	Reports of fatigue, aches, and side effects, or sadness or happiness, excitement, and gratefulness.	Infertility specialists recommended to partner with mental health specialists and avoid misinformation by using data.
Peterson and Buday (2020) (61)	To examine the effects of infertility on sexual relationships	One open-text question added to a quantitative survey	=202 adults from the St. Louis, MO metropolitan area who had one or more infertility consultations	Reported some discomfort in having sex to comply with IVF protocol or clinic's schedule.	ND	Couples described feeling relief in not having to pressure sex given switch to IVF technology.	ND	Encouraged couples to discuss sexual rights during IVF process to avoid sexual coercion.
Sira et al. (2024) (62)	To understand experiences of childhood cancer survivors now faced with infertility	Cross-sectional in-depth interviews	=6 adult childhood cancer survivors	Ambiguity in both healthcare provider terminology and in optional steps to evaluate fertility caused stress.	ND	Family support was a primary driver in choices made. Infertility was a stressor in current and prospective romantic relationships.	Discussed identity confusion caused by infertility and efforts to reframe negative situations productively and positively.	Survivorship programs and follow-up clinics should provide practical support (information and relationship and psychosocial support)
Wagi et al. (2022) (63)	To identify gaps in access to ART services	Cross-sectional in-depth interviews	=5 female graduate students seeking care for infertility	Often had to advocate for oneself with clinics to receive preferred ART/IVF treatment plan.	ND	Welcomed rare opportunity to talk to others with similar fertility experiences.	Experienced stress from view that infertility support is not needed for students.	Encouraged university infertility and pre-conception education programs and campus groups.

US, United States; DC, Washington DC; CA, California; MO, Missouri; ND, assessment domain was not discussed; ART, assisted reproductive technology; IVF, *in vitro* fertilization; OB/GYN, obstetrician/gynecologist; PCP, primary care physician.

### Quality assessment

As part of the study appraisal, the methodological rigor of each article's qualitative methods was also assessed Table 3). We adapted Jennings & Gagliardi's 10-item quality assessment checklist for use in the current review (64). The checklist item “Report of intervention implementation detail to facilitate replication” was removed as it is not relevant for the current review's inclusion criteria. The checklist evaluated whether the study included: prolonged engagement in a study setting, justification for design and methods selected, justification of the sampling strategy, description of analytical methods, use of verification methods to demonstrate credibility, provision of a reflexivity account, detailed report of findings, balanced representation of viewpoints, or presentation of conclusions supported by the data. Each quality item was scored on a binary scale, with 0 indicating the absence of the quality item and 1 indicating the presence of the quality item. Studies that received ≤4 points were categorized as having weak quality compared to moderate quality (5–6 points) or high quality (7–9 points).

**Table 3:** Distribution of qualitative quality criteria and overall rating for selected articles (*N* = 22).

Qualitative quality criteria	Number of selected articles with quality criteria	Percentage of selected articles with quality criteria (% = *n*/22)
1—Prolonged engagement in study setting	2	9%
2—Justification for design and methods selected	18	82%
3—Sampling strategy justified	18	82%
4—Analytical methods clearly described	18	82%
5—Use of verification methods to demonstrate credibility	5	23%
6—Reflexivity of account provided	3	14%
7—Detailed report of findings	20	91%
8—Balanced and fair representation of viewpoints	18	82%
9—Conclusions supported and confirmed by the data	19	86%
Overall qualitative quality rating		
Strong (7–9 points)	6	27%
Moderate (5–6 points)	11	50%
Weak (≤4 points)	5	23%

### Synthesis process

A content analysis approach was used to synthesize findings. First, we extracted and summarized findings by assessment domain for each article using the study appraisal form. Assessment domains were chosen to capture the scope of the literature based on an exploratory review and selected to inform future improvements in quality of care at the community and clinical levels (65, 66). To do so, all three reviewers (e.g., the first, second, and third authors) read each publication and its appraisal forms several times to interpret the summation of findings. These findings are reported by article row in Table 2. Next, we synthesized findings across applicable studies within each assessment domain by discussing connections between the article findings and each assessment domain. This synthesis of the findings for the total sample of articles by assessment domain is reported in the review's results section. A final step included summarizing implications for improving IVF care for adults in clinical or community settings in the U.S.

## Results

### Search and review process

Thirty-two (*n* = 32) full-text articles were retrieved for review from 1,682 potentially relevant citations based on the publication's title and abstract (Figure 1). The retrieved full-text articles were reviewed for eligibility, and ten (*n* = 10) were excluded. The most common reasons for exclusion were lack of assessment of experiences relating to IVF and lack of qualitative findings. We also excluded duplicates and publications that were not research articles, such as commentaries, protocols, editorials, or conference abstracts. An account of the number of exclusions is shown in Figure 1.

### Characteristics of selected studies

Twenty-two (*n* = 22) studies were retained in the final group of articles (42, 63). All the selected studies were pulled from an electronic database. The characteristics of the final set of studies are presented in Table 2. Although the search spanned over the past decade, most studies (*n* = 15; 68%) (42–44, 46, 47, 52, 53, 55–57, 59–63) were recently published within the last four and a half years (2020–2024). The majority of studies (*n* = 15; 68%) (44–46, 48–55, 57, 58, 62, 63) involved cross-sectional individual interviews with the exception of three studies (14%) that extracted qualitative online posts from social media (56, 59, 60), one study (5%) that involved repeated individual interviews (42), and three studies (14%) that used an open-text qualitative question that was added to a quantitative survey (43, 47, 61) (Table 2). Other qualitative methods such as focus group discussions or direct observations were not used. The qualitative sample sizes included small to large samples of 3–202 study participants in addition to 97–452 online qualitative posts (Table 2). All studies reported qualitative findings pertaining to at least one of the review's four assessment domains.

### Methodological quality

Table 3 summarizes the quality assessment of the selected articles. Six studies (27%) were rated as having strong qualitative methodological rigor (7–9 points). Eleven studies (50%) were rated as moderate (5–6 points), and five studies (23%) were rated as weak (≤4 points). We did not exclude studies with weak ratings given the limited number of relevant publications and the need to summarize findings. This is recommended for low-rated studies with no critical deficiencies (64). The most common methodological strengths were detailed report of findings with confirming quotations and conclusions supported and confirmed by the data (Table 3). The most common methodological weaknesses were absence of verification methods (e.g., member checking, triangulation, divergent case finding), omitted reflexivity, and lack of prolonged engagement, such as use of repeated or longitudinal inquiries.

### Health systems findings

Health systems findings, both positive and negative, were reported in the full set of included qualitative studies (*N* = 22), although most findings highlighted challenges in navigating IVF clinical systems (Table 2). Positive health systems findings were that patients found clinicians helpful in providing information and literature to understand the IVF process. They also valued living in communities with accredited fertility clinics given the challenge of transferring records and embryos. In some cases, patients also described appreciating mental health counseling referrals that were integrated into their IVF protocol. Despite these positive findings, the literature described numerous challenges in quality and continuity of care for individuals seeking or undergoing IVF. These challenges included feeling overwhelmed by the amount and type of clinical information, difficulty understanding information provided, feeling excluded in or misled by care decisions, disliking insensitive language used by clinicians, having difficulty managing multiple and uncoordinated providers and specialists, feeling that fertility concerns were invalidated by clinicians, and having difficulty obtaining timely diagnoses and care. Women were reported to be the most common initial contact to the health system for IVF usually with their primary care physician or OB/GYN who then referred them to a reproductive endocrinologist. They also relied on online patient data and reviews to select a fertility clinic or online forums to assist in interpreting IVF lab results. A few studies examined differences in health systems experiences for IVF patients due to COVID protocols, gender, age, or provider characteristics, although this was a less common approach.

### Economic findings

Economic experiences relating to IVF were assessed by about half of the included studies (*N* = 13), all describing the cost of IVF as a negative experience and primary barrier to utilization (Table 2). IVF patients reported using their credit cards and savings, taking out loans, or selling their belongings to cover care costs, which varied by state or metropolitan area. Some individuals regretted their lack of financial preparation for IVF or regretted not spending money earlier in life on fertility preservation (e.g., cryopreservation or egg freezing). Others reported difficulty understanding the potential costs and worry about whether insurance would cover any of the cost of treatment. In contrast, the literature also showed that some IVF patients struggled to rationalize whether the payments they made were worth it, including attributing decisions to discontinue IVF to high costs. Still others’ limited knowledge of their financial options (e.g., insurance, co-pays, loans) were described as a barrier to seeking care. The current literature also showed that individuals experienced additional financial strain when paying for complementary medical procedures (e.g., supplements, acupuncture, counseling) to cope with and supplement IVF. A few studies reported differences in economic experiences of IVF patients, noting that individuals from higher-income households could initiate IVF treatment more quickly or that clinicians sometimes made assumptions about one's ability to pay based on race or socio-economic status. A final economic finding was that IVF patients were concerned about the profit interests of clinics who encouraged paying patients to continue treatment even if their chance of success was low.

### Interpersonal and social findings

Interpersonal IVF experiences were almost always assessed in the qualitative literature (*N* = 20) and included concurrently affirming and non-affirming encounters (Table 2). For example, friends and families were characterized as important sources of social support, but were also reported as sources of stress from negative feedback, unwanted advice, and limited knowledge on how to help. Individuals also found it burdensome to have to educate their friends and family about IVF. A complaint was also how little infertility was discussed in participants’ current or former social circles. For this reason, the literature often noted that individuals undergoing IVF received the most support from people who had prior IVF experiences, especially women. Counseling and support groups were also described as positive opportunities to learn more about IVF and to connect with others with similar experiences. Yet, for some individuals, the literature also reported reluctance to discuss IVF with others to maintain privacy. Complex experiences were most commonly described with sexual partners, where the ups and downs of IVF were experienced jointly—as a couple. Yet, sometimes, individuals were uncertain how to alleviate their partner's stress or found it difficult to express their feelings with their partner.

### Physical and emotional findings

Nearly all articles (*N* = 21) reported on physical and emotional effects of IVF (Table 2). The most common finding was that individuals undergoing IVF experience frustration, anger, fear, and sadness from their IVF experiences. Findings also suggested that emotional effects varied by gender. One study found that men felt emasculated by infertility diagnoses, while women felt alarmed and surprised given the dominant narrative on pregnancy prevention. Another study found that the partner of the woman undergoing IVF felt worried for her future physical health given the impact the treatment would have on his partner's body. While most articles described negative emotions, two studies discussed feelings of happiness, excitement, and gratefulness for the benefits provided by IVF. However, a lack of knowledge around mental health resource options was expressed as a contributing factor to the overall emotional experience. Physical findings centered on the difficulty managing pain, fatigue, or related IVF side effects.

### Authors’ suggestions to improve IVF care

Numerous suggestions were provided by authors to improve IVF care (Table 2). At the clinic level, suggested interventions included increased training of health care providers, provision of more information on IVF options and costs, changes in policies requiring infertility diagnosis or assignment, standardized referrals for mental health services, and encouragement of clinic attendance with partners, as applicable. At the community level, suggested interventions included sharing more stories of IVF experiences across various racial and socio-economic groups (to minimize stereotypes), educating friends and families about how to help IVF peers, and expanding sexual health education to include use of assisted reproductive technologies (ART). Many of these suggestions were also mentioned by study participants.

## Discussion

To our knowledge, this narrative review is the first to-date to examine the current qualitative evidence of IVF experiences among U.S. adults. Findings showed that individuals accessing and undergoing IVF encounter complex and intersecting positive and negative experiences when navigating the health system, interpersonal relationships, and economic, emotional, and physical treatment requirements. We found that, while the current qualitative literature has variable methodological quality, it has grown in recent years and provides several recommendations for potentially improving IVF care experiences in community and clinical settings.

Findings showed that interactions with the healthcare system, via clinics and practitioners, yielded mixed feelings about standard of care procedures and treatment. The cost-prohibitive nature of IVF and navigating variable insurance coverage were both sources of stress and barriers to treatment utilization. The review also found that accessing or undergoing IVF impacted friendships, families, and partner relationships the most. In few cases relationships were strengthened, but in most others unsolicited advice, insensitive language, and lack of understanding caused strain. Many of the included studies also reported on the physical (e.g., pain, medication side effects) and emotional (e.g., stress, negative affect) toll of IVF. The literature points to a need for improved IVF care experience in clinical settings, and increased support in both the clinical and community spheres. The authors of the included studies also provided potential solutions, including health care provider trainings (e.g., trauma-informed care, provider sensitivity trainings), increased information dissemination, and experience sharing in clinics and the community.

While much of the literature centers around heterosexual women, particularly White women, and their experiences with IVF, one encouraging finding was the query of men and people of color on their experiences with IVF in the analyzed sample. However, we didn't find any literature centered around non-binary individuals, same-sex couples, or single (i.e., unpartnered) individuals, which is an area for future research. Understanding how ethical issues concerning malpractice and negligence in the IVF setting [e.g., failure to fully genetically test donor sperm (67), switching patient embryos (68)] impact patient-provider relationships, patient trust in the healthcare system, and fertility outcomes is an important area for further exploration. Future studies should also further examine the impact of location of intervention delivery, with the increasing reliance on digital spaces (i.e., social media) for information acquisition in addition to clinics and doctors’ offices. Finally, given the overturning of Roe v. Wade and its implications for reproductive and fertility care in the US, future research should explore the impact of healthcare access policy on IVF care experiences.

### Limitations

The limitations of this study should be considered. The search process was limited to studies that were published in English and that were available in peer-reviewed databases. As a result, findings may not be transferrable to non-English-speaking communities or representative of studies published in the gray literature. Additionally, the search was limited to studies that contained search strategy terminology in the title or abstract, leaving potentially relevant literature unidentified. However, strengths of this review include use of a systematic approach to identify, appraise, and synthesize findings of studies, review by multiple members of the study team, assessment of multiple topical domains, review of methodological rigor, and inclusion of the last 10 years of literature on IVF experiences.

## Conclusion

The qualitative literature of IVF experience necessitates more research specifically aimed at improving access, care experience, and social support in the health system and economic realms, and at the community level through interpersonal relationships. Currently, findings suggest that the experience of those accessing or undergoing IVF is fraught with stressors, from cost of treatment and complementary therapies to interactions with health care providers, friends, and family. In tandem with more research, increased education is urgently needed to begin de-stigmatizing the use ARTs and promoting, rather than hindering, positive social support and interactions.

## Data Availability

The data analyzed in this study is subject to the following licenses/restrictions: the submitted systematic review focuses on qualitative studies and all data analyzed have been previously published in other peer-reviewed journals. Additionally, no primary data are being made available with this manuscript. Requests to access these datasets should be directed to corresponding authors of included manuscripts. All manuscripts included in the review are cited in-text and in the references.
